# Data on the power of the creatinine to uromodulin ratio in serum to predict cardiovascular events in coronary patients

**DOI:** 10.1016/j.dib.2017.03.003

**Published:** 2017-03-09

**Authors:** Andreas Leiherer, Axel Muendlein, Christoph H. Saely, Janine Ebner, Eva M. Brandtner, Peter Fraunberger, Heinz Drexel

**Affiliations:** a Department of Medicine and Cardiology, Academic Teaching Hospital Feldkirch, Feldkirch, Austria; b Vorarlberg Institute for Vascular Investigation and Treatment (VIVIT), Feldkirch, Austria; c Private University of the Principality of Liechtenstein, Triesen, Liechtenstein; d Medical Central Laboratories, Feldkirch, Austria; e Drexel College University of Medicine, Philadelphia, PA, USA

**Keywords:** Uromodulin, Tamm–Horsfall-Protein, Creatinine, Ratio, Biomarker, Kidney disease, Cardiovascular events, Coronary patients, Mortality

## Abstract

Uromodulin is a protein which is produced by the tubular cells of the thick ascending limb in the kidneys and the creatinine to uromodulin ratio in serum recently has attracted interest as a marker of kidney disease. Whether this ratio also is associated with cardiovascular event risk is unknown. This article provides additional data on the association of the creatinine to uromodulin ratio with its power to predict cardiovascular events and major cardiovascular events in coronary patients. In addition, this data article demonstrates the performance of the creatinine to uromodulin ratio as a biomarker using c-statistics. Analyzed data was derived from 529 coronary patients. Uromodulin and creatinine were measured and cardiovascular events were recorded for up to 8 years. This data article is related to a research article titled “Serum Uromodulin is a predictive biomarker for cardiovascular events and overall mortality in coronary patients” [1].

**Specifications Table**

**Table:** 

Subject area	*Medicine, Clinical Research*
More specific subject area	*Cardiology, Epidemiology, Biomarkers*
Type of data	*Figures, table*
How data was acquired	*ELISA*
Data format	*Analyzed data*
Experimental factors	*Uromodulin and creatinine concentration in 529 coronary patients has been determined and cardiovascular events/major cardiovascular events have been recorded for up to 8 years*
Experimental features	*Uromodulin and creatinine in serum sample was measured by ELISA*
Data source location	*Feldkirch, Austria*
Data accessibility	*Data is with this article*
Related research article	*Leiherer, A., Muendlein, A., Saely, C., Ebner, J., Brandtner, E., Fraunberger, P., and Drexel, H., 2016. Serum Uromodulin is a predictive biomarker for cardiovascular events and overall mortality in coronary patients. Int.J.Cardiol.*http://dx.doi.org/10.1016/j.ijcard.2016.12.183.

**Value of the data**

– No prospective data on the power of the ratio between serum creatinine and serum uromodulin to predict cardiovascular events were available at present.
– This data article further proves the association of the creatinine to uromodulin ratio in serum samples with future cardiovascular events as described in the main article [1] by providing additional cox regression models.
– Whereas the main article [1] has evaluated the performance of uromodulin as a biomarker to predict mortality, this data article contains the c-statistics-based evaluation of the creatinine to uromodulin ratio to predict cardiovascular events and major cardiovascular events.
– This data article helps researchers to evaluate the potential of creatinine to uromodulin ratio as a biomarker.
– These data are important, because the biological role of uromodulin in blood is still elusive and may stimulate future research on uromodulin.

## 1 Data

**Fig. 1:**
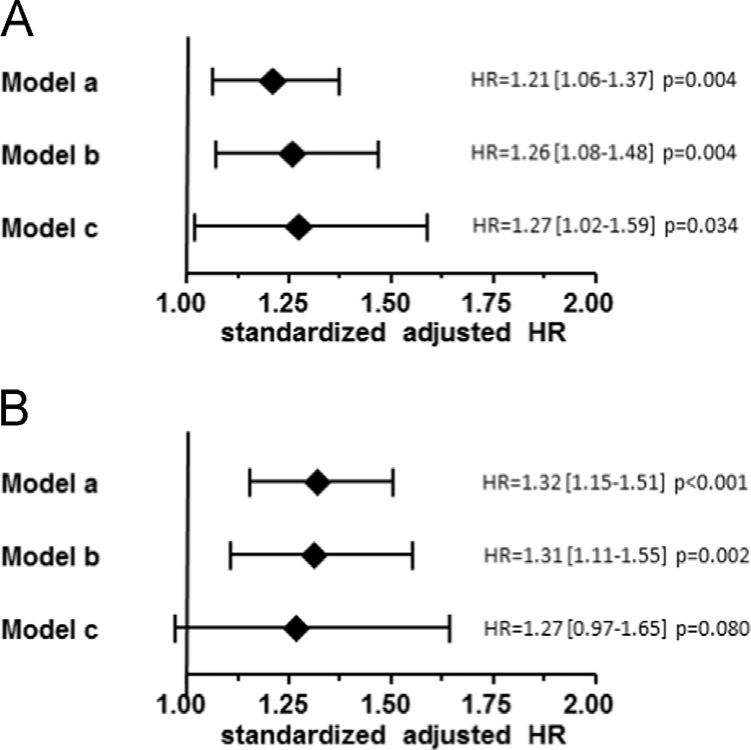
The creatinine to uromodulin ratio in serum as predictor of risk for cardiovascular events and major cardiovascular events. The Forest plot represents the adjusted hazard ratios (HR) with 95% confidence interval (CI) for the association between the creatinine to uromodulin ratio and the risk for cardiovascular events (A) and major cardiovascular events (B) in the study population during 6.5 years of FU. Study endpoint A comprises all cardiovascular events, including coronary death, fatal ischemic stroke, non-fatal myocardial infarction, non-fatal ischemic stroke, and need for coronary artery bypass grafting, percutaneous coronary intervention, or revascularization in the carotid or peripheral arterial beds. Study endpoint B comprises only major cardiovascular events, including coronary death, fatal ischemic stroke, non-fatal myocardial infarction, and non-fatal ischemic stroke. Model a includes the covariates age, gender, and body mass index (BMI). Model b includes the parameters included in model a and in addition systolic blood pressure (SBP), diastolic blood pressure (DBP), high density lipoprotein (HDL) and low density lipoprotein (LDL) cholesterol, the type 2 diabetes (T2DM) status, the current smoking status, c-reactive protein (CRP), the neutrophile-lymphocyte ratio (NLR), pro brain natriuretic protein (proBNP) and the CAD status. Missing values for SBP, DBP, HDL and LDL cholesterol, and proBNP were imputed by multiple imputation. Model c includes all parameters also included in model b but comprises no imputed data.

It has been mentioned in the main article [1] that there is a significant association between the creatinine to uromodulin ratio in serum of 529 coronary patients and the risk for cardiovascular events. Here, further adjustment models are provided demonstrating the predictive power of the creatinine to uromodulin ratio in serum with the risk for (A) cardiovascular events and (B) major cardiovascular events during follow up time (Fig. 1).

**Fig. 2:**
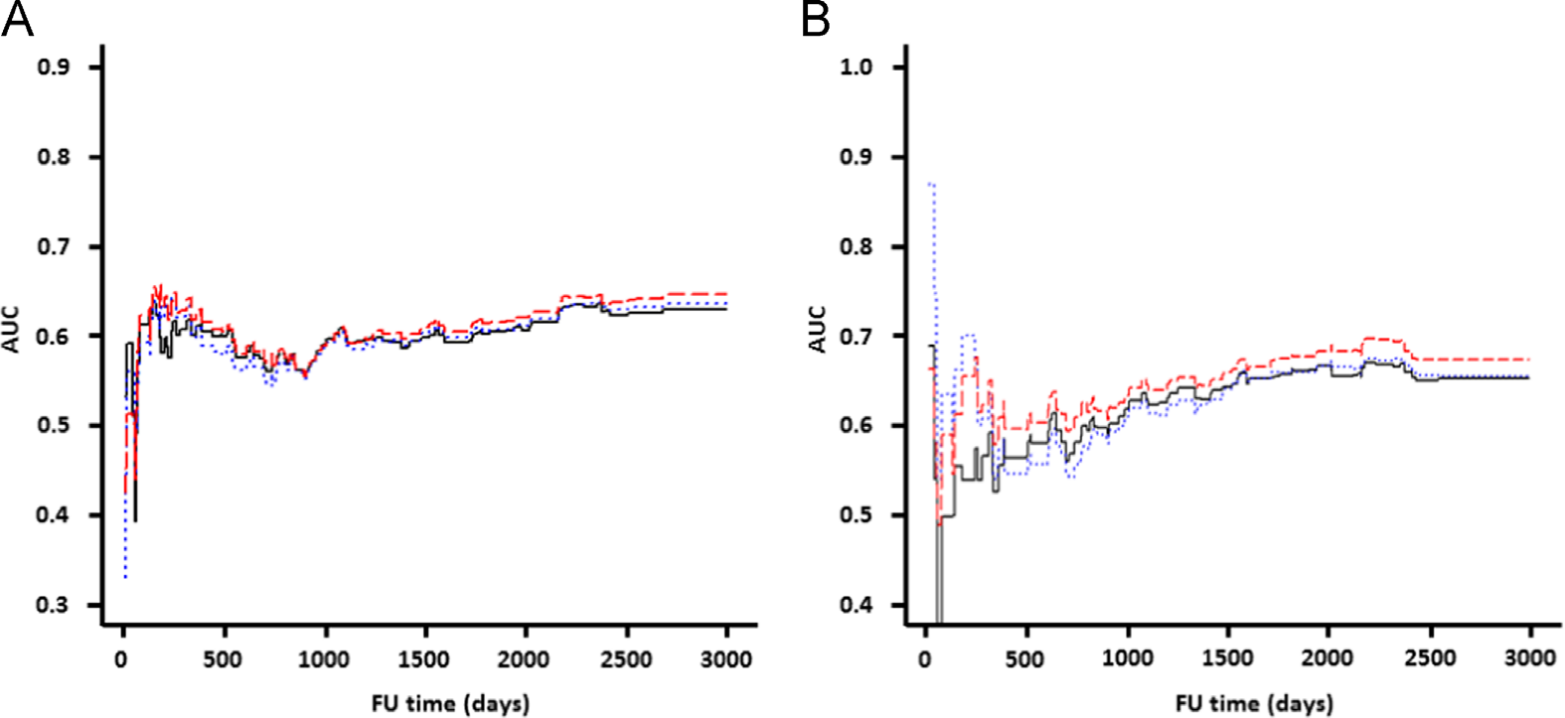
Area under the curve (AUC) over time based on prediction models of cardiovascular events (a) and major cardiovascular events (b) during follow up time. The plot represents the AUC for the prediction model “basic” (black solid line), “basic+ratio” (red dashed line), and “basic+creatinine” (blue dots), as described in Table 1*during the full follow up (FU) time.*

The data summarized in Table 1 show that the prediction of cardiovascular events and major cardiovascular events is significantly higher applying an enhanced prediction model comprising the creatinine to uromodulin ratio compared to a basic model lacking the creatinine to uromodulin ratio. In contrast, an alternative prediction model comprising only creatinine did not significantly improve prediction of cardiovascular events if compared to a basic model without creatinine. The performance of all prediction models over the FU time for cardiovascular events and major cardiovascular events is depicted in Fig. 2.

**Table 1:** C-statistics-based biomarker evaluation for cardiovascular events in models with and without the creatinine to uromodulin ratio.

Outcome	Model	*n*	*R*^2^	AUC	Harrell׳s C	Somers’D	IDI	*p*-value
A	Basic	508	0.036	0.628	0.602	0.203	–	–
	Basic+ratio	508	0.046	0.637	0.612	0.224	0.030	0.040
	Basic+creatinine	508	0.045	0.635	0.609	0.217	0.024	0.109
B	Basic	508	0.038	0.648	0.635	0.270	–	–
	Basic+ratio	508	0.054	0.666	0.654	0.307	0.036	0.050
	Basic+creatinine	508	0.058	0.655	0.641	0.282	0.043	0.040

The first model comprises age, systolic blood pressure (SBP) diastolic blood pressure (DBP), the current smoking status, low density lipoprotein cholesterol (LDL) and the diabetes mellitus type 2 status (T2DM) and was determined basic. The basic model (basic) was compared to models additionally comprising, creatinine (basic+creatinine) or the creatinine to uromodulin ratio (basic+ratio). Models were built as linear predictor scores after cox regression. The area under the curve (AUC) for the receiver operator characteristic (ROC) is given for cardiovascular events (outcome A) and for major cardiovascular events (outcome B). Integrated discrimination index (IDI) for the addition of the creatinine or the creatinine to uromodulin ratio to the basic model is given with respective p-values at *t*=2900 days. The IDI for the use of the ratio added to the basic model, instead of creatinine added to the basic model was 0.005 (*p*=0.667) for outcome A and −0.007 (*p*=0.557) for outcome B.

## 2 Experimental design, materials and methods

### 2.1 Study design and analyses

The 529 coronary patients were recruited between 2005 and 2008. Characterization and basic laboratory measurement was done as described in the main article [1]. In short, only patients who were referred to elective coronary angiography for the evaluation of established or suspected stable CAD were enrolled. Patients undergoing coronary angiography for other reasons and patients with acute coronary syndromes were excluded. Serum creatinine concentrations were measured using the modified Jaffé method (Creatinine Jaffé Gen.2 Assay, Roche, Basel, Switzerland). Serum uromodulin levels were determined with a commercial uromodulin enzyme-linked immunosorbent assay (ELISA) kit (BioVendor, Brno, Czech Republic; catalog no. RD191163200R), specific for human uromodulin with an inter-assay variation <6.5%. Patients had a mean (±standard deviation) serum uromodulin concentration of 164.9 (±77.2) ng/ml and a mean serum creatinine concentration of 0.888 (±0.249) mg/dl. The ratio between serum creatinine (mg/dl) and serum uromodulin (ng/ml) in the population ranged from 7.89e^−4^ to 5.03e^−2^.

The study׳s mean follow up time (±standard deviation) was 6.5 (±1.8) years and the follow up rate was 98%. The study endpoint “cardiovascular event” was a composite of coronary death (fatal myocardial infarction, sudden cardiac death, mortality from congestive heart failure due to CAD), fatal ischemic stroke, non-fatal myocardial infarction non-fatal ischemic stroke, and need for coronary artery bypass grafting (CABG), percutaneous coronary intervention (PCI), or revascularization in the carotid or peripheral arterial beds. Coronary angioplasty and bypass surgery were considered as end points unless they were planned as a consequence of the baseline angiography and therefore were not “future” events. The study endpoint “major cardiovascular event” (which may also be referred to as major adverse cerebro-/cardiovascular event), was a composite of coronary death (fatal myocardial infarction, sudden cardiac death, mortality from congestive heart failure due to CAD), fatal ischemic stroke, non-fatal myocardial infarction, and non-fatal ischemic stroke.

### 2.2 Statistical analysis

Statistical analyses as described in this data article were performed with SPSS 21.0 for Windows (SPSS, Inc., Chicago, IL) and R statistical software v. 3.2.3 (http://www.r-project.org) and are described in detail in the main article [1]. In particular, to determine the incidence of the respective endpoints, we used Cox proportional hazards models with z-transformed continuous variables. To examine the potential utility of the creatinine to uromodulin ratio as a predictive biomarker [2], several cox regression models were fitted with the respective study endpoint as the dependent variable and c-statistics were applied. The respective models were compared according to their linear predictor score using calculation of Harrell׳s C and Somers’ D. Time-dependent receiver operating curves (ROC) and the respective area under the curve (AUC) were calculated using the survivalROC package for R applying nearest neighbor estimation (NNE) method as described elsewhere [3]. The IDIs with respective p-values were calculated using the survIDINRI package [4], [5]. All missing values were missing completely at random (MCAR) according to Little׳s MCAR test. We used the Markov Chain Monte Carlo (MCMC) method with Predictive Mean Matching (PMM) as multiple imputation method to estimate the missing data.
